# Supplementary material for: End Sequence Analysis Toolkit (ESAT) expands the extractable information from single-cell RNA-seq data
Source: Genome Res. 2016 Oct;26(10):1397–410. doi: 10.1101/gr.207902.116 (PMC5052061; doi:10.1101/gr.207902.116)
Supplement: Supplemental Material [file supp_gr.207902.116_Supp_Materials.tar.gz › ESAT-esat_v0.1/doc/html/classbroad_1_1core_1_1siphy_1_1_pi_h_m_m.html]

ESAT: broad.core.siphy.PiHMM Class Reference


ESAT


- Main Page
- Related Pages
- Packages
- Classes
- Files

- Class List
- Class Index
- Class Hierarchy
- Class Members

All Classes Namespaces Files Functions Variables Enumerator Pages

- broad
- core
- siphy
- PiHMM

Classes |
Public Member Functions |
Static Public Member Functions |
Static Public Attributes |
Protected Member Functions |
List of all members

broad.core.siphy.PiHMM Class Reference

Inheritance diagram for broad.core.siphy.PiHMM:

[legend]

Collaboration diagram for broad.core.siphy.PiHMM:

[legend]

|  |  |
| --- | --- |
| Classes | |
| class | **ConstrainedModel** |
|  | |
| class | **PiStateModel** |
|  | |

|  |  |
| --- | --- |
| Public Member Functions | |
|  | PiHMM (EvolutionaryModel neutral, int stateNumber) |
|  | |
|  | PiHMM (EvolutionaryModel neutral, double minExpectedElementLength, double expectedCoverage) |
|  | |
| void | addConservedPi (Matrix pi, String name) |
|  | |
| short[] | viterbiMostLikelyEstimation (MultipleAlignment alignment) throws BadModelException |
|  | |
| double | computePathLogLikelihood (MultipleAlignment alignment, short[] path, int initialStateIdx, int endStateIdx) |
|  | |
| ForwardResult< Map< String,   Matrix > > | runForwardAlgorithm (MultipleAlignment alignment) throws BadModelException |
|  | |
| BackwardResult< Map< String,   Matrix > > | runBackwardAlgorithm (MultipleAlignment alignment, ForwardResult< Map< String, Matrix >> forwardData) throws BadModelException |
|  | |

|  |  |
| --- | --- |
| Static Public Member Functions | |
| static void | main (String[] args) throws Exception |
|  | |
| static PiHMM | createDefaultTwoStateChain (double l, double gamma, EvolutionaryModel model) |
|  | |
| static PiHMM | createTwoStateChain (double l, double gamma, EvolutionaryModel model, Matrix otherPi) |
|  | |

|  |  |
| --- | --- |
| Static Public Attributes | |
| static final String | USAGE |
|  | |

|  |  |
| --- | --- |
| Protected Member Functions | |
| MultipleAlignment | generateAlignment (List< Integer > emittedPath, String format) |
|  | |

## Constructor & Destructor Documentation

|  |  |  |  |
| --- | --- | --- | --- |
| broad.core.siphy.PiHMM.PiHMM | ( | EvolutionaryModel | *neutral*, |
|  |  | int | *stateNumber* |
|  | ) |  |  |

Here is the caller graph for this function:

|  |  |  |  |
| --- | --- | --- | --- |
| broad.core.siphy.PiHMM.PiHMM | ( | EvolutionaryModel | *neutral*, |
|  |  | double | *minExpectedElementLength*, |
|  |  | double | *expectedCoverage* |
|  | ) |  |  |

## Member Function Documentation

|  |  |  |  |
| --- | --- | --- | --- |
| void broad.core.siphy.PiHMM.addConservedPi | ( | Matrix | *pi*, |
|  |  | String | *name* |
|  | ) |  |  |

|  |  |  |  |
| --- | --- | --- | --- |
| double broad.core.siphy.PiHMM.computePathLogLikelihood | ( | MultipleAlignment | *alignment*, |
|  |  | short[] | *path*, |
|  |  | int | *initialStateIdx*, |
|  |  | int | *endStateIdx* |
|  | ) |  |  |

|  |  |  |  |
| --- | --- | --- | --- |
| static PiHMM broad.core.siphy.PiHMM.createDefaultTwoStateChain | ( | double | *l*, |
|  |  | double | *gamma*, |
|  |  | EvolutionaryModel | *model* |
|  | ) |  |  |

static

Here is the call graph for this function:

Here is the caller graph for this function:

|  |  |  |  |
| --- | --- | --- | --- |
| static PiHMM broad.core.siphy.PiHMM.createTwoStateChain | ( | double | *l*, |
|  |  | double | *gamma*, |
|  |  | EvolutionaryModel | *model*, |
|  |  | Matrix | *otherPi* |
|  | ) |  |  |

static

Here is the call graph for this function:

|  |  |  |  |
| --- | --- | --- | --- |
| MultipleAlignment broad.core.siphy.PiHMM.generateAlignment | ( | List< Integer > | *emittedPath*, |
|  |  | String | *format* |
|  | ) |  |  |

protected

|  |  |  |  |  |  |
| --- | --- | --- | --- | --- | --- |
| static void broad.core.siphy.PiHMM.main | ( | String[] | *args* | ) | throws Exception |

static

Here is the call graph for this function:

|  |  |  |  |
| --- | --- | --- | --- |
| BackwardResult<Map<String, Matrix> > broad.core.siphy.PiHMM.runBackwardAlgorithm | ( | MultipleAlignment | *alignment*, |
|  |  | ForwardResult< Map< String, Matrix >> | *forwardData* |
|  | ) |  | throws BadModelException |

|  |  |  |  |  |  |
| --- | --- | --- | --- | --- | --- |
| ForwardResult<Map<String, Matrix> > broad.core.siphy.PiHMM.runForwardAlgorithm | ( | MultipleAlignment | *alignment* | ) | throws BadModelException |

|  |  |  |  |  |  |
| --- | --- | --- | --- | --- | --- |
| short [] broad.core.siphy.PiHMM.viterbiMostLikelyEstimation | ( | MultipleAlignment | *alignment* | ) | throws BadModelException |

## Member Data Documentation

final String broad.core.siphy.PiHMM.USAGE

static

**Initial value:**

= "Usage: PiHMM TASK=<task\_num> <task\_args>\n" +

"\tTasks:\n" +

"\t\t1. Segment genome into pi-conserved and noncoserved. -in <multiple alignment file> -mod <Neutral Evolutionary model consisting of aminoacid background distribution, mutation matrix and neutral phylogenetic tree>" +

"\n\t\t -l <The smoothness parameter, and should be the expected average conserved element length> and -gamma <The expected coverage parameter, how much of the genome is expected to be conserved>" +

"\n\t\t -format <Alignment format default is FASTA is default> -ignore <comma separated species to ignore> -ref <reference sequence id, necessary if the alignment is not in MAF format>" +

"\n\t\t [-start <If MAF file, you may specify the reference start coordinate> -end <If MAF file, you may specify the reference end coordinate>]" +

"\n\t\t2. Compute posterior probabilities of each site of being pi-conserved. -in <multiple alignment file> -mod <Neutral Evolutionary model consisting of aminoacid background distribution, mutation matrix and neutral phylogenetic tree>" +

"\n\t\t -l <The smoothness parameter, and should be the expected minimum conserved element length> and -gamma <The expected coverage parameter, how much of the genome is expected to be conserved>" +

"\n\t\t -format <Alignment format default is FASTA is default> -ignore <comma separated species to ignore> -ref <reference sequence id, necessary if the alignment is not in MAF format>" +

"\n\t\t [-start <If MAF file, you may specify the reference start coordinate> -end <If MAF file, you may specify the reference end coordinate>]" +

"\n\t\t3. Compute log odds score for annotations in file. Scores reflect the log ratio of the probability of the path through each element being fully conserved or non conserved. -in <Annotation file default format is assumed to be BED> -mod <Neutral Evolutionary model consisting of aminoacid background distribution, mutation matrix and neutral phylogenetic tree>" +

"\n\t\t -l <The smoothness parameter, and should be the expected minimum conserved element length> and -gamma <The expected coverage parameter, how much of the genome is expected to be conserved>" +

"\n\t\t -format <Alignment format default is FASTA is default> -ignore <comma separated species to ignore> -ref <reference sequence id, necessary if the alignment is not in MAF format>" +

"\n\t\t [-start <If MAF file, you may specify the reference start coordinate> -end <If MAF file, you may specify the reference end coordinate>]" +

"\n\t\t4. Simulate an alignment with for given HMM parameters. \n\t\tParameters:\n\t\t-mod <Base m,odel to use> \n\t\t-ignore <optional -- comma separated species to ignore in the given model tree> \n\t\t-colNum <Number of columns to sample> -\n\t\t-otherPI <Conserved or alternative PI distribution, as a comma separated list of the A,C,G,T frequencies, add as many -otherPI as different PI states are desired. > " +

"\n\t\t -l <The smoothness parameter, how long in average should be the stretches of the generated alignment should be from the alternative PI model> and -gamma <The coverage parameter, how much of the sampled alignmnet should be made of the alternative PI model> \n\t\t-out <Generated alignment output file>" +

"\n"

---

The documentation for this class was generated from the following file:

- java/broad/core/siphy/PiHMM.java


---

Generated on Fri Aug 15 2014 15:10:57 for ESAT by  

 1.8.7
